# Association of Mycoplasma Pneumoniae Infections with Status Asthmaticus

**DOI:** 10.2174/1874306400802010035

**Published:** 2008-05-07

**Authors:** Usama Hanhan, James Orlowski, Mariano Fiallos

**Affiliations:** University Community Hospital, Tampa, FL 33613, USA

**Keywords:** Mycoplasma pneumoniae, asthma, status asthmaticus, chest X-ray infiltrates.

## Abstract

**Background & Objective:**

Viral respiratory infections (VRI) have been commonly associated with exacerbation of wheezing in asthmatic children. Mycoplasma pneumoniae (MP) causes many respiratory syndromes that clinically mimic VRI. Due to the nature of the organism, cultures are of no practical value and the diagnosis is usually made by serology. Only a few studies have associated mycoplasma infections with acute exacerbations of wheezing in the asthmatic patient. This study was an attempt to assess the incidence of recent mycoplasma infections in patients with status asthmaticus and to review their laboratory, clinical and radiological findings.

**Methods:**

Retrospective review of all patients admitted to PICU over 12 month period with status asthmaticus. Recent mycoplasma infection was determined utilizing the Immunocard Mycoplasma Enzyme Immunoassay (EIA) for detection of MP IgM antibodies (Meredian Diagnostics, Inc., Cincinnati, OH)

**Results;:**

The records of 44 patients were reviewed. 9 were excluded because MP tests were never obtained during hospitalization. 15/35 (42%) were MP Positive. There were no statistically significant differences (P>0.05) in length of hospitalization (LOH), ICU days, duration of continuous albuterol aerosol hours (cont. Nebs hrs.), days on O2 (02 days) or WBC between the two groups, however patients who were mycoplasma positive were treated with a macrolide antibiotic in addition to their standard asthma therapy. Patients with evidence of recent MP infection were more likely to have one or more infiltrates on their CXR (13/15 *vs* 7/20; P= 0.002).

**Conclusion:**

Our study suggests that recent MP infections play a significant role in exacerbations of asthma and occurrence of status asthmaticus in children. The presence of infiltrates on CXR in status asthmaticus warrants tests for MP.

## INTRODUCTION

Status asthmaticus is among the leading causes of pediatric admissions to the hospital. Viral respiratory infections have been commonly associated with exacerbations of asthma in children [1]. Only a few studies have associated mycoplasma infections with acute exacerbations of wheezing in the asthmatic patient [2-5]. In addition to being the most common cause of community acquired atypical pneumonia in children, Mycoplasma pneumoniae (M.P.) causes other respiratory syndromes such as bronchitis, bronchiolitis, pharyngitis and croup [6,7]. Many of these syndromes mimic viral respiratory infections in children. Due to the nature of the organism and its very slow growth rate, cultures have been of no practical clinical value and the diagnosis is usually made by serological methods [8].

It has been our belief, as well as others, that recent mycoplasma infections play a much greater role in the pathophysiology of asthma and the precipitation of status asthmaticus than is readily recognized. We also suspected that infections with mycoplasma were often overlooked and not treated, and often times the infection was termed a “viral infection.” This study was an attempt to assess the incidence of recent mycoplasma infections in patients with status asthmaticus that were admitted to our Pediatric Intensive Care Unit and to review their laboratory, clinical, and radiological findings.

## METHODS

The medical records of all patients less than twenty years old with the diagnosis of status asthmaticus between September 1997 and October 1998 were obtained. Status asthmaticus was defined as failure to respond to the usual appropriate initial emergency room treatment that included at least three albuterol aerosols, and necessitating PICU admission. Demographic, clinical and laboratory data were reviewed including age, sex, duration of asthma, hospital length of stay, ICU days, duration of oxygen treatment, duration of continuous albuterol aerosol treatments, mycoplasma IgM, initial radiological findings on chest x-ray and WBC count. Two groups of patients were identified: MP positive and MP negative patients. Comparison among variables between the two groups were then analyzed statistically by Chi square and Students’t-test. All patients suspected of having a respiratory tract infection in association with their asthma exacerbation were tested for Mycoplasma pneumoniae utilizing the Mycoplasma IgM rapid test on admission and the results were available within 2-4 hours. Patients that tested positive were started on a macrolide antibiotic in addition to the standard asthma treatment. All patients received systemic steroids as part of their therapy. Patients in whom the Mycoplasma IgM test was not obtained were excluded from the study. Chest x-rays were reviewed by a radiologist blinded to the results of the Mycoplasma IgM test, and were considered positive if they demonstrated the presence of one or more infiltrates.

Mycoplasma IgM rapid tests: The Immunocard Mycoplasma Enzyme Immunoassay (EIA) (Meredian Diagnostics, Inc., Cincinnati, Ohio) is a qualitative procedure for the detection of IgM to mycoplasma pneumoniae. This methodology provides a simple to use, self-contained assay. No calculations are required and the visual color change makes interpretation of the results objective and simple. The test cards are individual foil pouches containing immobilized detergent extracted Mycoplasma pneumoniae antigens and human IgM. Included also are positive/negative controls, enzyme conjugate, wash buffer, and substrate reagents. The manufacturer’s directions were followed to perform the test in our laboratory. Performance characteristics of the Immunocard mycoplasma tests as published by the manufacturer showed a relative sensitivity of 88% (± 6%), relative specificity of 90% (± 3%), and agreement of 90% (± 3%) when compared with a microwell EIA. Discrepant results were resolved by Immunofluorescent Assays (IFA), latex and complement fixation testing (CFT).

## RESULTS

There were 44 patients with a diagnosis of status asthmaticus admitted to the PICU during the study period. 9 patients were excluded because the mycoplasma IgM test was never obtained during the hospital period. Among the remaining 35 patients, 15 (42%) were mycoplasma IgM positive. Age range of the mycoplasma positive patients was 2-19 years (mean 9.4 years) and there were 8 males and 7 females. 20 patients (58%) were mycoplasma IgM negative, 14 males and 6 females with an age range of 1.5 to 19 years (mean of 7.5). There were no statistically significant differences (P greater than 0.05) between the two groups in terms of length of hospitalization (LOH), ICU days, duration of continuous albuterol aerosols (Cont. Nebs Hrs.), days on oxygen (O_2_ days), or white blood cell count (Table **1**). Patients who were mycoplasma positive were treated with a macrolide antibiotic in addition to their standard asthma therapy which included continuous albuterol nebulization at 0.3 mg/kg per hour, atrovent aerosols and systemic steroids.

13 (86%) of the 15 patients with positive mycoplasma IgM tests had a positive chest x-ray (infiltrates) on presentation *vs* 7 (35%) of the 20 patients with negative mycoplasma IgM titers (P value =0.002). One of our patients had multiple admissions during the study period, was initially mycoplasma IgM negative on December 27, 1997 and January 25, 1998 and then became positive on one admission on February 18, 1998 and then negative subsequently on admission in April 1998. Her chest X-ray showed a left lower lobe infiltrate in February 1998 and hyperinflation with no infiltrates on the April 1998 admission.

## DISCUSSION

Acute exacerbation of wheezing in the asthmatic patient in association with respiratory tract infections has been well documented. Several studies have linked viral upper and lower respiratory infections as common precipitants of acute asthma exacerbations. Our study revealed an incidence of 42% of recent Mycoplasma pneumoniae infections among pediatric asthmatic patients admitted with acute exacerbation of their asthma and status asthmaticus. Recent infection was suggested by the presence of positive qualitative mycoplasma IgM against Mycoplasma pneumoniae.

Only a few studies have addressed the incidence of Mycoplasma pneumoniae infections in asthmatic patients. In a series of 84 children with asthma, Berkovich *et al. *[3] in 1970 demonstrated a 32.1% incidence of viral or Mycoplasma pneumoniae infections during exacerbations of their disease. In their study significant changes in antibodies to Mycoplasma pneumoniae were found in 7 of the patients. (3 had evidence of Mycoplasma pneumoniae infection alone while 4 had Mycoplasma pneumoniae combined with a virus.) Gil *et al. *[9] in a study of 77 patients with asthma and 88 persons without asthma (controls) demonstrated that Mycoplasma pneumoniae was isolated from 24.7% of asthmatic patients compared to 5.7% of the control group, demonstrating a significantly higher colonization rate in the asthmatic patient. Yano *et al. *[10] demonstrated in a case report the onset of bronchial asthma symptoms following a recent mycoplasma infection. They also found the presence of IgE antibodies specific to Mycoplasma pneumoniae in that patient. Tipirneni *et al. *[5] detected IgE antibodies to Mycoplasma pneumoniae in 5 of 152 patients with asthma and other atopic diseases. Seggev *et al. *[2], in a study of 95 adult patients (mean age of 45.7 years) hospitalized due to acute asthma, demonstrated that 21% of these patients had evidence of a recent Mycoplasma pneumoniae infection. Other studies [11] have also suggested a role for Chlamydia pneumoniae infections in association with both acute and chronic wheezing.

In contrast to these studies, others have not found evidence of Mycoplasma pneumoniae infections among adults and children with acute asthma. Minor *et al. *[12] in their study found an increased incidence of viral URIs during exacerbations of asthma but no evidence of Mycoplasma pneumoniae. Berman *et al. *[13] in a study based exclusively on cultures of trans-tracheal aspirates, found no evidence of any infection associated with exacerbations of asthma.

Our study does demonstrate a high incidence of recent mycoplasma infections as demonstrated by a positive mycoplasma IgM test. This along with other studies suggests a much greater role for mycoplasma infections in the acute exacerbation of asthma. We were unaware of any epidemic of Mycoplasma pneumoniae in our area during the time of the study period.

Direct detection of Mycoplasma pneumoniae infections by culture is currently difficult because of the slow growth rate of the organism and fastidious growth requirements. For this reason, serology is often the best laboratory method available. Various formats are available to test patients’ sera to Mycoplasma pneumoniae. Complement fixation detects IgM and IgG, and although it has been used extensively in the past, recent evidence suggests that it lacks specificity. Similarly, cold agglutinin serology is both insensitive and non-specific in children younger than 12 years of age, and many experts have recommended against requesting cold agglutinins for pediatric age groups [7]. On the other hand, enzyme-linked immunosorbent assays (ELISA) are sensitive and adaptable and most of the serologic tests used now are based on the ELISA format. Although obtaining paired sera for the detection of IgM and IgG in the acute and convalescence phases of an infection would usually establish the diagnosis during convalescence and would be useful to assess prevalence of past infections, most clinicians would like to confirm the diagnosis early during the acute infection. Therefore serological determination of current mycoplasma infection by a single, acute-phase serum sample often is the only diagnostic test used in routine clinical care. Some disadvantages to this method, however, are that even though specific IgM antibodies to Mycoplasma pneumoniae are usually detected in patients with a recent primary infection, they may be found in patients with reactivated or secondary infections and are sometimes found in patients with no other detectable evidence of recent infection (unapparent infections.) Utilizing IgM serology, however, will help detect most recent and some past Mycoplasma pneumoniae infections and therefore clinical correlation in interpreting the results is important. Similarly, Mycoplasma pneumoniae can be recovered by culture or PCR from the respiratory tract up to several weeks after an acute infection often times despite antibiotic use, making the interpretation of those results difficult as well (colonization versus acute or chronic infection). Even after accounting for the limitation of the Immunocard Mycoplasma EIA test that we used in our study (sensitivity 88% - specificity 90%, and 90% agreement when compared with a microwell EIA), it is still apparent that mycoplasma infections are frequently seen in association with acute asthma exacerbations. Our study also found no significant differences in length of hospitalization, ICU stay, days on oxygen, continuous albuterol aerosol requirements, or WBC, between mycoplasma positive and mycoplasma negative patients. However, it should be noted that all of our patients who were found to be mycoplasma IgM positive were treated with a macrolide antibiotic in addition to their standard asthmatic treatment within six hours of admission to the hospital. Whether such antibiotic treatment had a beneficial and positive effect on the clinical progress of these patients is yet to be determined.

One significant and important finding of this study is the frequency of finding a positive Chest X-ray (infiltrates) in those asthmatics who were Mycoplasma IgM positive. All chest x-rays were reviewed by a radiologist blinded to the results of the Mycoplasma IgM test, and were considered positive if they showed one or more definite infiltrates. Although infiltrates or variable opacifications are not uncommon in asthmatic patients and may represent atelectasis from mucous plugging rather than infection, the difference between the two groups was very significant. (13 of the 15 patients who were mycoplasma positive versus 7 of 20 patients who were Mycoplasma negative, P=0.002). This finding would suggest that the presence of one or more infiltrates in patients with status asthmaticus warrants testing for the presence of recent mycoplasma infection. The Mycoplasma EIA Immunocard test costs $227.00 for a kit of 28 tests including mandatory controls.

Our study has a number of limitations. It was a retrospective study, the diagnostic test does not differentiate with absolute certainty recent from remote or reactivated Mycoplasma infections, but neither does the PCR or the cultures. Other important infectious agents associated with status asthmaticus such as viruses, were not examined in our study. Nevertheless we found a high incidence of mycoplasma infections in our patients presenting with status asthmaticus.

In a recently published study, Kraft *et al. *[14] detected the presence of Mycoplasma pneumoniae by PCR in bronchoalveolar lavage and biopsy specimens in 10 of 18 chronic stable asthmatics (55%) and in 1 of the 11 control subjects, suggesting the presence of Mycoplasma pneumoniae in the lower airways of chronic stable asthmatics with significantly greater frequency than in the control non-asthmatic subjects. All of the patients, however, who had the organism detected by polymerase chain reaction (PCR) had negative cultures, EIAs and serology for Mycoplasma pneumoniae in this study. The authors in their discussion state that PCR positivity can be present for longer periods than culture or seropositivity. They hypothesize that these asthmatic patients are chronically infected or colonized with Mycoplasma pneumoniae.

In a related study Martin *et al. *[15] extended their initial report to include a total of 55 chronic stable asthmatics and 11 normal control subjects. In this study 25 of the 55 asthmatics had positive PCR results for Mycoplasma and 6 for Chlamydia, strongly suggesting that both Mycoplasma pneumoniae and Chlamydia are found in asthmatic airways. Furthermore they noted the presence of increased numbers of mast cells in the group with positive PCR results suggesting a potential interaction between chronic infection and allergen sensitization. Again as with the original study, all cultures and serology for Mycoplasma were negative in both asthmatics and control groups.

Despite our understanding of the histopathology of mycoplasma infections and its attachment to the ciliated respiratory epithelial cells, it remains unclear what mechanism this bacteria employs to cause injury and harm to the respiratory cells. It is also unclear whether it triggers an immune- mediated response that causes bronchospasm. Whether Mycoplasma pneumoniae plays a significant role in the pathogenesis of both acute and chronic asthma is yet to be determined. If in fact it does, then treatment of selected groups of asthmatics with anti-mycoplasma antibiotics might prove to be beneficial.

## CONCLUSION

Although the association of the respiratory infections with the exacerbation of asthma has been described in the literature, as we alluded in our discussion, only a few studies have shown an association of Mycoplasma pneumoniae infections with asthma and especially with status asthmaticus. Also an equal number of studies have failed to show any association of Mycoplasma pneumoniae infections with exacerbation of asthma.

We think our study adds new information especially relevant to pediatric practice because we addressed status asthmaticus in children. Mycoplasma pneumoniae is an important organism associated with status asthmaticus and acute exacerbation of asthma in children and the presence of infiltrates on chest x-ray in status asthmaticus warrants testing for Mycoplasma pneumoniae.

Whether treating Mycoplasma pneumoniae positive patients with macrolide antibiotic is beneficial or not is something we are addressing in a subsequent study.

## Figures and Tables

**Fig. (1).**
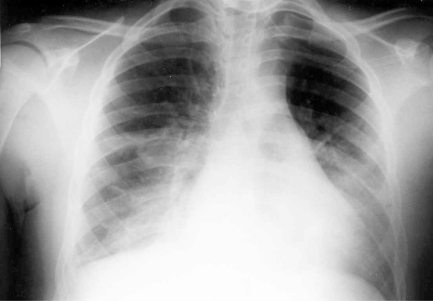
Chest X-ray of a patient in status asthmaticus who is Mycoplasma pneumoniae positive demonstrating bilateral basilar infiltrates.

**Table 1.** Characteristic of Patients with Status Asthmaticus

	Pts	Sex	*Mean Age (Yrs)	LOH (Days)	ICU (Days)	O_2 _Days	Cont Nebs (Hrs)	WBC	** + CXR
**MP +**	15	8 M	9.4	5.2	2.75	3.5	27.7	15.2	13
**MP -**	20	14 M	7.5	4.65	2.65	3.35	29	13.6	7
**P- value**	NS	NS	NS	NS	NS	NS	NS	NS	0.002

* Age Range 2-19 years.

M = Male.

** Presence of one or more infiltrates.

LOH = Length of hospitalization.

ICU = Length of ICU stay.

O2 days = Duration of requirement for supplemental oxygen

Cont Nebs = Duration of requirement for continuous nebulized albuterol.

+ CXR = Chest X-ray abnormal.
